# Pentraxin 3 Detects Clinically Significant Fibrosis in Patients with Chronic Viral Hepatitis C

**DOI:** 10.1155/2019/2639248

**Published:** 2019-04-02

**Authors:** Joanna Gorka-Dynysiewicz, Monika Pazgan-Simon, Jolanta Zuwala-Jagiello

**Affiliations:** ^1^Department of Pharmaceutical Biochemistry, Wroclaw Medical University, Poland; ^2^Department of Infectious Diseases and Hepatology, Wroclaw Medical University, Poland

## Abstract

Pentraxin 3 (PTX3) plays a pathogenic role in experimental models of chronic liver injury and contributes to the progression of fibrosis. The detection of advanced fibrosis (METAVIR F≥3) is important to identify patients who are in urgent need of antiviral treatments* versus* those whose treatment could be deferred (F≥2). The aim was to assess the diagnostic value of PTX3 as a potential biomarker for clinically significant and advanced fibrosis. PTX3 associations with biochemical and histological parameters of inflammatory activity and fibrosis were investigated in 138 patients with chronic viral hepatitis C (HCV) before antiviral treatment. METAVIR histological scores of activity and fibrosis were obtained. PTX3 was measured by enzyme-linked immunosorbent assay. The diagnostic accuracy of serum PTX3 levels was compared to that of other fibrosis markers, including transforming growth factor‐*β*_1_ (TGF-*β*_1_), hyaluronic acid (HA), aspartate transaminase to platelet ratio index (APRI), fibrosis score based on four factors (FIB4), gamma-glutamyltranspeptidase to platelet ratio (GPR), and the liver stiffness measurement (LSM) by transient elastography (FibroScan®). In HCV patients the PTX3 level increased in parallel with the METAVIR histological score of activity, being independently associated with the METAVIR fibrosis score (*P* < 0.001). Using the receiver operating characteristics analysis, the best marker for detecting F≥2 and F≥3 was PTX3 with AUC = 0.802 and AUC = 0.867, respectively. The area under the curve of PTX3 for predicting significant fibrosis (F≥2) was significantly greater than those for the GPR ratio (AUC = 0.648) and FIB-4 score (AUC = 0.770) and similar to that for APRI index (AUC = 0.831). PTX3 provided clinically relevant diagnostic accuracy as a single marker of significant fibrosis.

## 1. Introduction

Chronic viral hepatitis C (HCV) is characterized by a progression of fibrosis, which ultimately leads to the formation of cirrhosis. The management of chronic hepatitis depends on the degree of liver fibrosis. Thus, the assessment of the degree of liver fibrosis is important for choosing a therapeutic strategy and for determining the prognosis. Liver biopsy is the gold standard method for evaluating the degree of liver fibrosis [1]. However, the invasive nature of liver biopsy makes it impractical, especially for patients who require follow-up [2]. Therefore, many reports have demonstrated noninvasive examination methods for assessing the degree of fibrosis in chronic liver disease, which may be alternatives to liver biopsy, such as new serum biomarkers or biomarker panels and transient elastography [3–5].

Pentraxin 3 (PTX3) is an essential component of the humoral arm of innate immunity, involved in the resistance against microorganisms and inflammation. It is well established that key activators of the inflammatory and reparative response after tissue injury, such as proinflammatory cytokines, damaged tissue-derived signals, and microbial molecules, induce PTX3 production in different cell types, including vascular endothelial cells, mesenchymal cells, and fibroblasts, as well as in cells of innate immunity, such as monocytes and granulocytes. Neutrophils store PTX3 in secondary granules and upon appropriate stimulation promptly release it. In a simplistic view, PTX3 plays a crucial role in inflammation through activation of the complement cascade and inhibition of P-selectin-mediated neutrophils extravasation. Since PTX3, an acute-phase protein rapidly produced by vascular tissues as well as by cells of innate immunity in response to inflammation, is a rapid and reliable marker for primary local activation of innate immunity and inflammation in several disorders, including acute kidney injury, severe acute respiratory syndrome or LPS-induced lung injury, atherosclerosis, and acute myocardial infarction [for more detail, see review [6]].

Recently, Doni et al. [7], in agreement with previous reports [8–11], have shown a nonredundant protective role of PTX3 in the regulation of tissue repair and remodeling. In various murine models of tissue damage, including skin-wound healing, chemically induced sterile liver, and lung injury and arterial thrombosis, PTX3 deficiency was associated with increased clotting as well as with fibrin and collagen deposition/persistence, epithelial hyperplasia, and defective mature tissue formation at healing. This phenotype was attributed to the lack of PTX3-dependent facilitated plasmin-mediated fibrinolysis by tissue remodeling cells, which is a prerequisite for appropriate tissue repair [7]. In this respect, fibrin and other provisional extracellular matrix proteins are deposited after tissue injury and their subsequent timely degradation is essential for tissue repair [12, 13].

As mentioned above, PTX3 participate in the wound healing response in different organs, including the liver [7]. The experiments in cell showed that while PTX3 is mainly expressed in neutrophils in healthy liver; in injury it is mainly expressed in hepatic stellate cells (HSCs) [14], the main cell type responsible for the reparative response after tissue injury and liver fibrosis [15]. The differential expression of PTX3 in healthy and injured liver together with the data that show that PTX3 drives HSC activation [14] suggest that in response to injury PTX3 released by neutrophils may promote HSC activation. However, in chronic liver disease, activated HSCs would become the main cell type producing PTX3, which may enhance the reparative response after liver injury and exert protective and immune modulatory effects. In light of these observations, further clinical studies are needed to elucidate the precise role of the PTX3 in liver fibrosis.

Close associations between PTX3 levels, disease progression, and the stages of liver fibrosis in patients with nonalcoholic fatty liver disease and alcoholic hepatitis were shown [14, 16, 17]. Only one study concerning the PTX3 polymorphism [18] analyzed the ability of serum PTX3 levels to predict liver fibrosis in HCV patients. The authors decided not to include significant fibrosis (F≥2) group in the study to ensure more homogeneous phenotype. To our knowledge, ours study is the first one to examine the diagnostic accuracy and characteristics of serum PTX3 levels in HCV patients with significant (F≥2) and advanced (F≥3) fibrosis.

We hypothesized that the measurement of PTX3 could provide an alternative to the existing noninvasive serum markers to assess the disease stage in a cross-sectional study of HCV patients. The primary aim was to evaluate PTX3 to identify HCV patients who were in urgent need of antiviral treatments (METAVIR fibrosis F≥3) and those whose treatment could be deferred (METAVIR fibrosis F≥2). A secondary aim was to compare the diagnostic value of PTX3 to validated fibrosis markers, including serum markers, such as hyaluronic acid (HA), transforming growth factor‐*β*1 (TGF-*β*1), aspartate aminotransferase (AST) to platelet index (APRI), FIB‐4 score, *γ*-glutamyltranspeptidase (GGT) to platelet ratio (GPR), and liver stiffness measurement by transient elastography. The study found that PTX3 provided clinically relevant diagnostic accuracy as a single marker of liver fibrosis.

## 2. Patients and Methods

### 2.1. Patients

The study cohort consisted of 178 adults, including 40 healthy volunteers, and 138 patients with chronic hepatitis C, who were admitted to the Department of Infectious Diseases and Hepatology from October 2015 to December 2017. The diagnosis of chronic hepatitis C infection was based on persistently increased alanine aminotransferase values, anti-HCV and HCV-RNA positivity and liver histology features. The HCV inflammation was confirmed by measuring HCV-Ab and HCV-RNA in the serum with the use of the EIA methods and RT PCR, Cobas Amplicor Roche methods, respectively. Patients dually infected with HCV and HBV or patients with fatty liver, which might influence the value of FibroScan®, were excluded. Patients with known substance (alcohol and/or intravenous drugs) abuse as well as those with HIV (human immunodeficiency virus), autoimmune or congenital metabolic liver conditions, malignancies, or being treated with immunosuppressants were excluded from the study, as well. None of the patients received antiviral therapy prior to inclusion. The purpose of each examination was fully explained, and informed consent was obtained from all participants. The study protocol was approved by the ethics committee of the Bioethics Committee of the Wroclaw Medical University and carried out in accordance with the 1975 Declaration of Helsinki (revised in 2008).

### 2.2. Liver Histology and Quantification of Liver Fibrosis

Liver biopsy was performed as part of the workup to assess the severity of inflammation and fibrosis. Stained biopsies were examined by one expert pathologist blinded to patient clinical characteristics and scored according to the METAVIR scoring system: F0–F4 for the degree of fibrosis (F0, no fibrosis; F1, portal fibrosis alone; F2, portal fibrosis with rare septa; F3, portal fibrosis with many septa; F4, cirrhosis) and A1–A3 for the degree of necroinflammatory activity (A1, mild activity; A3, marked activity). The presence of F2, F3, or F4 stages was referred to as significant fibrosis (F≥2), while the presence of F3 or F4 stages was referred to as advanced fibrosis (F≥3). All patients classified as METAVIR F4 has compensated disease.

### 2.3. Clinical and Laboratory Assessment

The sample of peripheral venous blood from fasted patients with chronic hepatitis C was taken on the same day; as liver biopsy procedures and FibroScan® examination were carried out blood was allowed to clot for 30 min at 25°C and centrifuged at 2000×g for 15 min at room temperature; then serum was separated and aliquoted into tubes for storage. The tubes were frozen at −80°C and stored in order to be used for the analysis of different parameters. The concentrations of alanine transaminase (ALT), aspartate transaminase (AST), *γ*-glutamyltransferase (GGT), total bilirubin, serum albumin, platelets, leukocytes, cholesterol, HDL-cholesterol, triglycerides, hyaluronic acid, and international normalized ratio (INR) were measured using standard clinical methods. The Model for End-Stage Liver Disease (MELD) score was calculated using bilirubin, creatinine, and INR [19]. The aspartate aminotransferase (AST) to platelet index (APRI), FIB-4 score, and GPR ratio were calculated based on the following formulae:

APRI index = (AST (IU/L)/upper normal limit) x 100/platelets (10^9^/L) [20];

FIB−4 score = age (years) x AST (IU/L)/platelets (10^9^/L) x (ALT (IU/L))^1/2^) [21];

GPR ratio = GGT (IU/L)/platelets (10^9^/L), where GGT is *γ*-glutamyltransferase [22].

PTX3 levels were determined with the use of the ELISA method (*Cloud-Clone Corp., Houston, USA*) by following the instructions in the manual provided by the manufacturer. An ELISA plate reader (model* Multiscan*™* Go* microplate reader,* Thermo *Scientific™*, Finland*) was used to measure the color intensity according to the instructions in the manual provided by the manufacturer, and the PTX3 level of each sample was determined. Control samples and serum standards with concentrations that ranged from 0.312 to 20.0 ng/mL were included in each run. The minimum detectable dose of PTX3 was typically less than 0.113 ng/mL. Serum levels of HA were measured using latex agglutination turbidimetry (*Echelon Biosciences, Salt Lake City, USA*). TGF-*β*_1_ concentrations were measured with the use of the immunoenzymatic method (*Diaclone SAS, Besancon Cedex, France*).

### 2.4. Measurement of Liver Stiffness by Transient Elastography, FibroScan®

Transient elastography (FibroScan® Echosens, Paris) was performed by a skillful operator to assess the LSM (liver stiffness measurement) value. LSMs by transient elastography were expressed in kilopascals (kPa) and were evaluated in relation to the interquartile range (IQR) and success rate of measurements. The IQR of less than 30% of mean liver stiffness and the success rate of more than 60% in more than 10 validated measurements were indicative of a successful measurement. According to Tsochatzis [23] et al., the liver stiffness cut-offs were presented on a scale of 0–4 according to FibroScan® given as 7.6 (range 5.1–10.1), 10.5 (8.0–15.4), and 15.3 (11.9–26.5) kPa for stages F2, F3, and F4 in chronic hepatitis C patients, respectively.

### 2.5. Statistical Analysis

Continuous variables are expressed as median (interquartile range; IQR) or mean ± standard deviation and categorical variables as number (percentage). We performed one-way analysis of variance (ANOVA) to evaluate whether PTX3 was useful to judge the METAVIR fibrosis score or not. Afterward, we examined the correlation of PTX3 with liver function parameters and other conventional fibrosis markers using the Spearman rank correlation. For logistic regression analyses, the *P* value of each independent variable was determined by the Wald chi-square value (Wald), which was calculated by squaring the ratio of the regression coefficient divided by its standard error. The diagnostic value of PTX3 for predicting significant (F≥2) and advanced fibrosis (F≥3) or cirrhosis (F=4) was assessed by calculating the areas under the receiver operating characteristics (ROC) curves. ROC curves were generated by plotting the sensitivity against 1 − specificity, and the area under the curve (AUC) with 95% confidence intervals (95% CI) was calculated. According to DeLong [24], the empirical nonparametric method was performed to make pairwise comparisons of ROC curves. Based on the ROC analysis the optimum cut-off point was established by selecting the value that provides the greatest sum of the sensitivity and specificity, i.e., the point closest to the upper left point of the ROC plot. For the optimum cut-off point provided by each ROC analysis, the sensitivity, specificity, positive predictive value (PPV), and negative predictive value (NPV) were calculated using standard formulas. Statistical significance was defined as *P* < 0.05. Statistical analyses were performed using* Statistica version 13.3* and* R version 3.5.1.*

## 3. Results

### 3.1. Patient's Demographic and Clinical Characteristics and Biochemical Data

Basic clinical and biochemical data for HCV patients and healthy controls are presented in Table 1. In the case of HCV patients, the median age was 55.0 years with a male predominance (60.1%), while the median age in the control group was 47.0 years with a female predominance (55%). METAVIR fibrosis scores in HCV patients were F0 (24 patients; 17.4%), F1 (25 patients; 18.1%), F2 (39 patients; 28.3%), F3 (21 patients; 15.2%), and F4 (29 patients; 21%); the median PTX3 level was 4.8 ng/mL (IQR, 1.01-12.7 ng/mL). In HCV patients, who underwent the FibroScan® test, the fibrosis stage was < F2 (LSM< 7.6) for 47 cases (34.1%), F2 or F3 (7.6 ≤ LSM< 15.3) for 61 cases (44.2%), and F4 (LSM > 15.3) for 30 cases (21.7%). As far as healthy controls are concerned, the median PTX3 level was 0.96 ng/mL (IQR, 0.20–1.96 ng/mL). In patients with chronic hepatitis C, the values of other fibrosis markers were hyaluronic acid (HA) at a median of 113.5 ng/mL (IQR, 7.9-826.9 ng/mL); transforming growth factor‐*β*_1_ (TGF-*β*_1_) at a median of 8.0 ng/mL (IQR, 2.12–31.5 ng/mL); APRI index at a median of 0.69 (IQR, 0.20–12.1); FIB-4 score at a median of 3.43 (IQR, 0.28–30.6); AST/ALT ratio at a median of 0.91 (IQR, 0.45–2.70); and GPR ratio at a median of 0.31 (IQR, 0.40-6.62) (Table 1).

### 3.2. Pentraxin 3 in Histological Scores of Inflammatory Activity and Fibrosis in Patients with Chronic Hepatitis C

**Figure 1:**
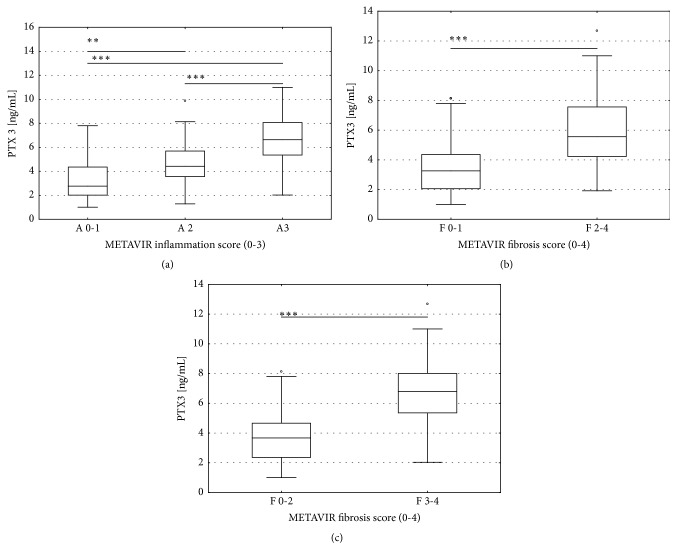
PTX3 in histological scores of inflammatory activity and fibrosis in patients with chronic hepatitis C. (a) PTX-3 and METAVIR inflammation score (0-3); ^*∗∗*^*P* < 0.01 between scores of inflammatory activities A0-A1 and A2; ^*∗∗∗*^*P* < 0.001 between scores of inflammatory activities A0-A1 and A3 and A2 and A3; (b) PTX3 and METAVIR fibrosis score (0-4); ^*∗∗*^*P* < 0.01 between fibrosis stages F0-F1 and F≥2; (c) ^*∗∗∗*^*P* < 0.001 between fibrosis stages F0-F2 and F≥3. The top and bottom of each box represent the first and third quartiles, respectively, with the height of the box representing the interquartile range, covering 50% of the values. The line across each box represents the median. The whiskers show the highest and lowest values.

**Figure 2:**
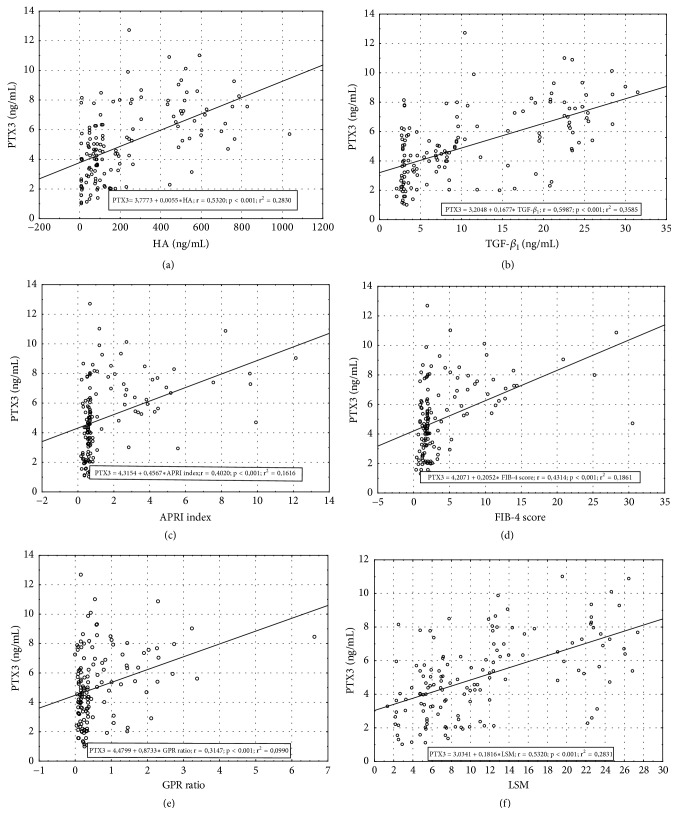
Correlation between PTX3 levels and (a) hyaluronic acid (HA), (b) TGF-*β*_1_, (c) APRI index, (d) FIB‐4 score, (e) GPR ratio, and (f) LSM. Data were analyzed by Spearman's rank correlation coefficient test. APRI, platelet ratio index; FIB‐4 score; GPR, gamma-glutamyltranspeptidase to platelet ratio; HA, hyaluronic acid; LSM, liver stiffness measurement; TGF-*β*_1_, transforming growth factor‐*β*_1_.

PTX3 increased with an increase in scores of inflammatory activity (Figure 1(a)) and correlated with METAVIR inflammation score (rho = 0.58,* P* < 0.001) in HCV patients. PTX3 significantly correlated with the histological stage of liver fibrosis (rho = 0.64,* P* < 0.001), and the levels of PTX3 were significantly higher in patients with significant fibrosis (F≥2) compared to F0-F1 (*P *< 0.001) (Figure 1(b)). PTX3 was also significantly higher in patients with advanced fibrosis (F≥3) compared to F0-F2 (*P* < 0.001) (Figure 1(c)). We used ordered logistic regression analysis with METAVIR fibrosis score as the dependent variable and PTX3 and ten biochemical parameters as the explanatory variables. In the univariate analyses, natural logarithms of the serum PTX3 and TGF-*β*1 levels provided the most significant coefficients (Wald = 66.78,* P* <0.01 and Wald = 96.06,* P* <0.01, respectively) (Table 2). Since PTX3 was related to the histological stage of liver fibrosis in HCV patients, we investigated whether PTX3 correlated with validated fibrosis markers, including direct serum markers (hyaluronic acid (HA), transforming growth factor‐*β*_1_ (TGF-*β*_1_), and indirect markers, such as aspartate aminotransferase (AST) to platelet index (APRI index), FIB‐4 score, and gamma-glutamyltranspeptidase to platelet ratio (GPR ratio). Serum PTX3 levels were significantly correlated with HA (Figure 2(a)) and TGF-*β*_1_ (Figure 2(b)) (*P *< 0.001 each). There was also a significant correlation between PTX3 levels and indirect serum markers of liver fibrosis, including APRI index (Figure 2(c)), FIB-4 score (Figure 2(d)), and GPR ratio (Figure 2(e)) (*P* < 0.001 each). Additionally, using the Spearman rank correlation analysis, a positive correlation between LSM and PTX3 level (rho = 0.53,* P* < 0.001) was observed (Figure 2(f)). These results strongly suggest a significant association of PTX3 with the histological severity of liver fibrosis.

### 3.3. Comparison of AUCs and Cut-Off Values for Fibrosis Markers

**Figure 3:**
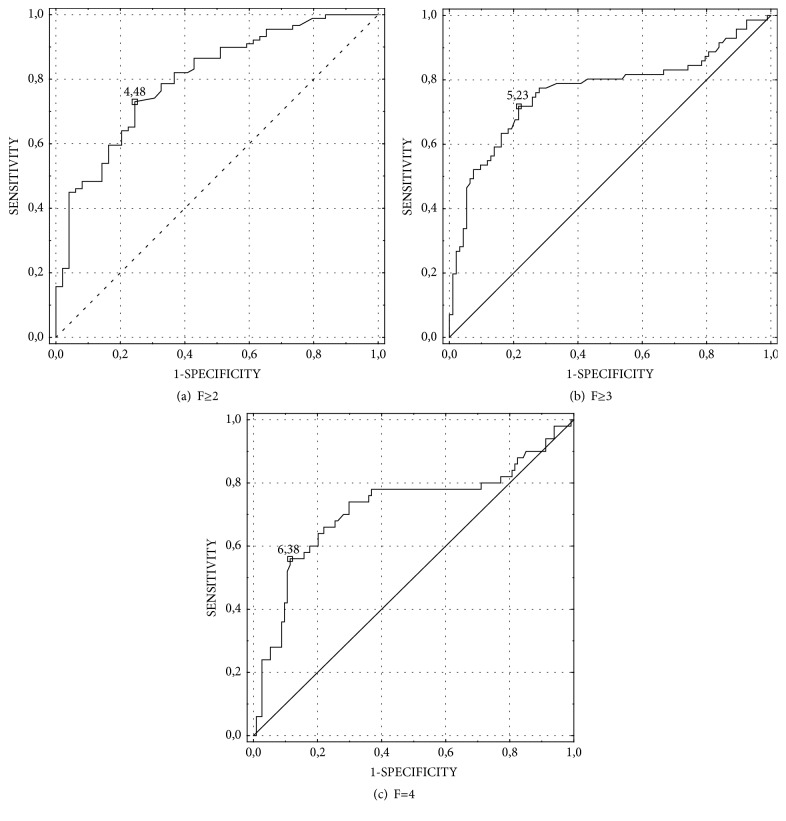
Diagnostic performances of PTX3 for the detection of significant (F≥2), advanced (F≥3) fibrosis, and cirrhosis (F=4) in patients with chronic HCV.

The area under the ROC curves was used to evaluate the diagnostic values for detecting significant (F≥2) or advanced (F≥3) fibrosis and cirrhosis (F=4) (Figure 3; Table 3). The best marker was PTX3 with AUCs = 0.802 for detecting F≥2 (Figure 3(a)), AUCs = 0.867 for detecting F≥3, and AUCs = 0.937 for detecting cirrhosis (Figure 3(c)). The optimal cut-off values for predicting fibrosis stages F≥2 and F≥3 and cirrhosis were 4.48, 5.23, and 6.38 ng/mL, respectively (Table 3).

**Figure 4:**
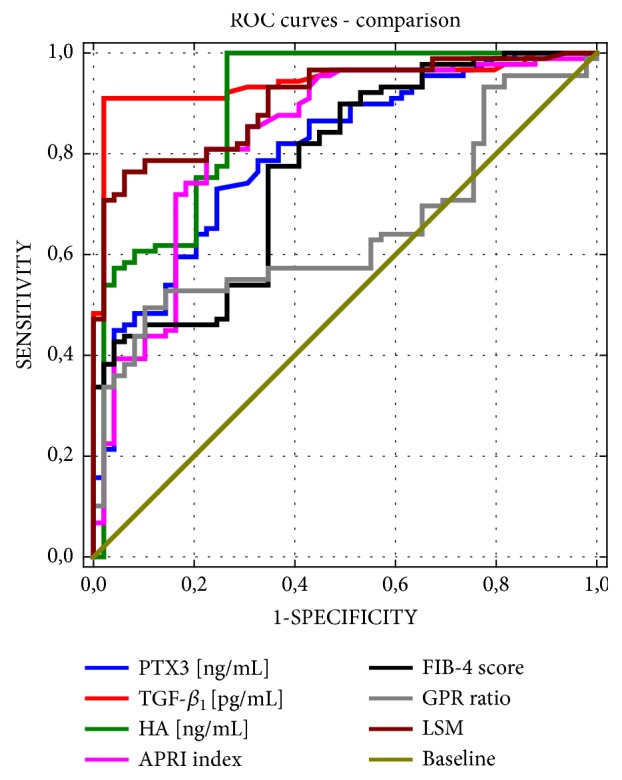
Comparison of PTX3 with other indicators, such as hyaluronic acid (HA), transforming growth factor‐*β*_1_ (TGF-*β*_1_), the aspartate aminotransferase (AST) to platelet ratio index (APRI index), the FIB‐4 score, gamma-glutamyltranspeptidase to platelet ratio (GPR ratio), and liver stiffness measurement (LSM), for the diagnosis of significant fibrosis (F≥2) by areas under the receiver operating curves (ROC).

The ROC curves for PTX3, TGF-*β*_1_, hyaluronic acid (HA), APRI index, FIB-4 score, GPR ratio, and LSM values for predicting significant fibrosis (F≥2) are shown in Figure 4. We analyzed the diagnostic accuracy of PTX3 levels to predict fibrosis stages F≥2 by ROC, and the area under the ROC was 0.802 (P<0.001) (Figure 4). The optimal cut-off point of PTX3 for predicting F≥2 was 4.48 ng/mL, and its sensitivity, specificity, positive predictive value (PPV), and negative and predictive value (NPV) were 73.0%, 75.5%, 84.4%, and 60.7%, respectively (Table 4). Using the DeLong method [24], the pairwise comparison of ROC curves was performed. To estimate significant fibrosis, the AUC for the PTX3 level (AUC = 0.802) was higher than that for the GPR ratio (AUC = 0.648,* P* < 0.01), but the differences were not significant and were comparable to that of the FIB-4 score (AUC = 0.770,* P* > 0.05), APRI index (AUC = 0.831,* P* > 0.05), and HA (AUC = 0.891,* P* > 0.05). Compared to PTX3, both TGF-*β*_1_ and LSM values were significantly better for detecting significant fibrosis (F≥2) (AUC = 0.943,* P* < 0.01; AUC = 0.904,* P* = 0.02, respectively) (Table 4). Moreover, our LSM cut-off for significant fibrosis (8.7 kPa) was close to this proposed in other studies [25, 26].

## 4. Discussion

Liver biopsy has been generally accepted as the most reliable method for evaluating the degree of liver fibrosis. However, it is an invasive procedure that cannot be carried out too often to follow the disease progression. Therefore, the field of noninvasive approaches for liver staging has recently evolved. Noninvasive markers such as serum markers or liver stiffness measurement (LSM, a significant indicator of liver stiffness as liver fibrosis) by transient elastography (FibroScan®) may be used to aid and/or replace liver biopsy to stage liver fibrosis [27–29].

The present study is the first one to evaluate the diagnostic accuracy and characteristics of serum PTX3 levels in HCV patients with significant (F≥2) and advanced (F≥3) fibrosis. The principal findings were as follows: (1) PTX3 provided clinically relevant diagnostic accuracy as a single marker for predicting fibrosis stages; (2) the accuracy of PTX3 levels for diagnosing significant (F≥2) liver fibrosis, measured as sensitivity, specificity, PPV, and NPV, was significantly higher than the accuracy for the GPR ratio (AUC = 0.648) and FIB-4 score (AUC = 0.770) and similar to that for the APRI index (AUC = 0.831) and HA (AUC = 0.891). PTX3 could be an alternative noninvasive serum marker for liver biopsy to assess liver fibrosis.

Our study clearly demonstrated that the PTX3 level in HCV patients increased with the progression of liver fibrosis stage. This complies with previous studies [16–18, 30], which show close associations between PTX3 levels, disease progression, and stages of liver fibrosis in patients with nonalcoholic steatohepatitis and/or alcoholic hepatitis and chronic viral hepatitis C. The results of our study further demonstrate that among ten variables, including PTX3 level, platelet count, albumin, AST, ALT, GGT, total bilirubin, INR, HA, and TGF-*β*1, both PTX3 and TGF-*β*1 (a direct fibrosis marker) were the most significant serum markers associated with severity of liver fibrosis. Although there was a positive correlation of PTX3 with the progression of liver fibrosis stage (rho = 0.64,* P* < 0.001) in our patients with HCV, the results from an* in vivo, ex vivo*, and* in vitro* studies [7, 10] suggest that PTX3 exerts a hepatoprotective and a modulatory effect on chronic inflammatory events, taking into account the fact that PTX3 protein and regulation are conserved between mice and humans [31]. One likely explanation of this apparently contradictory result is that PTX3 might be expressed as a strong evidence emerging from studies of Perea et al. [14] suggesting that the liver may be an important source of circulating PTX3. Indeed, it has been demonstrated that PTX3 hepatic gene expression and plasma levels showed a positive correlation in patients with alcoholic hepatitis and alcoholic cirrhosis. Independent of their etiology, the common hallmark of chronic liver diseases is chronic inflammation which shows a correlation with the progression of fibrosis. In the injured liver chemotactic stimuli trigger the rapid recruitment of immune cells including macrophages and neutrophils. These infiltrating immune cells then produce numerous proinflammatory cytokines and growth factors, which trigger the activation of myofibroblasts, the main effector cells of tissue remodeling [32]. Accordingly, there are reasons to believe that the increase in circulating PTX3 levels is likely triggered by ongoing production of proinflammatory cytokines at necroinflammatory injury, including IL-1 and TNF-*α*, which are known to be potent inducers of PTX3 release [33].

This phenomenon may also be reflected in the significant correlation between serum PTX3 levels and hepatic necroinflammatory activity (rho = 0.58, P < 0.001) in patients with HCV. In the early stages where fibrosis reduces PTX3 production may be more efficient than in the advanced fibrosis stages with more inflammation. On the contrary, in the CCL_4_-induced liver injury model, PTX3 deficiency did not affect necrosis; however, it was associated with an augmented fibrosis [7]. Therefore, further research is needed to clarify the molecular mechanisms of hepatic PTX3 production in HCV patients with different necroinflammatory activity grades.

Studies showed that several risk scores such as the APRI index, FIB‐4 score, and GPR ratio appeared to be a good surrogate marker for predicting fibrosis stages [20–22]. The AUCs of reported fibrosis markers (APRI index, FIB‐4 score, GPR ratio, HA, TGF-*β*1, and LSM values) increased as the liver fibrosis stages progressed; the AUC of PTX3 increased accordingly as well. The diagnostic performance of PTX3 was significantly better than that of the GPR ratio for detecting F≥2 with an AUC = 0.802. It is worth noting that PTX3 shows better diagnostic accuracy than the FIB-4 score, but with overlapping confidence intervals; therefore, it is not significantly different. Several studies evaluated the diagnostic performances of FIB-4 score and APRI index as noninvasive algorithms for detecting significant and/or advanced fibrosis [34–36]. They all obtained similar results as us, except for Wai et al., who found that the mean APRI AUC for significant fibrosis is 0.800 [20]. This APRI test showed that AUCs for detecting significant fibrosis ranged from 0.740 to 0.870, which was similar to the diagnostic performance of PTX3 (0.756-0.906). In light of these observations, results about the possible use of PTX3 as an early marker for predicting fibrosis stages are promising and further both preclinical and clinical studies are needed to elucidate the precise role of the PTX3 in the fibrosis.

There are three main strengths of this study. Firstly, the sample size (n = 138) was large enough and included well-determined baseline clinical characteristics. Although the number of patients enrolled might seem relatively small, it adequately represented the sample size estimated to provide the specific power. Secondly, in this study, we excluded patients with fatty liver disease, which may affect the LSM values. Thirdly, major strength of the study is the comparison to FibroScan® as this novel elastography technique fulfills a number of the requirements of an ideal noninvasive marker of fibrosis. However, FibroScan® has some limitations such as the lack of standardized cut-off for diagnosing fibrosis stages [32]. Because we relied on a single determination of PTX3, we cannot take into account any variation that may have occurred over time. However, this does not seem to be a major limitation because other fibrosis markers were measured only once in this study. Finally, liver biopsy is prone to sampling errors in the scope of the evaluation of the degree of liver fibrosis, leading to bias. Despite the fact that in this study PTX3 detects significant fibrosis with similar accuracy to that of indirect biomarkers APRI and FIB-4, PTX3 is still clinically relevant as it reflects fibrosis-related inflammation and not liver function such as APRI and FIB-4 calculation (AST and ALT values). Further, recent progress in antiviral treatment has normalized AST and ALT values in many patients even though they still have fibrotic liver [37]. In this context, PTX3 may be of more clinical relevance in terms of the general clinical practices related to the monitoring of patients whose treatment has been deferred to reconsider the indication of treatment and to discuss new therapies as they emerge.

In conclusion, we assessed the PTX3 for detection of clinical significant and advanced fibrosis in patients with chronic hepatitis C. PTX3 proved useful as single diagnostic marker. In the new study (a manuscript for an article is in preparation), based on our finding showing a significant correlation of the PTX3 value with the degree of liver fibrosis, we combined the PTX3 and HA or TGF-*β*1 values and defined a new index.

## Figures and Tables

**Table 1:** Clinical and biochemical characteristics of patients with chronic viral hepatitis C (HCV) and healthy controls.

	HCV patients	Healthy controls
(*n*)	138	40
Male Female, n (%)	(83:55), (60.14%: 39.86%)	(18:22), (45%:55%)
Age (years)	55 (22-79)	46 (18-67)
Etiology of hepatitis, (n) HCV-Ab(+)	138	-
HCV genotype, n (%)		
1b	100 (72.5%)	-
3a	29 (21.0%)	-
4c/4d	9 (6.5%)	-
HCV viral load, mean x 10^5^ copies/mL	2.84 (0.019 -7.13)	-
MELD score	7.6 (6.5-8.1)	-
BMI (kg/m^2^)	22.2 ±2.4	21.7 ±1.9
ALT (IU/L)	64 (13-278)**∗∗∗**	24.5 (10-38)
AST (IU/L)	50 (17-242)**∗∗∗**	27.5 (12-35)
ALP (IU/L)	82 (38-220)**∗∗**	65 (52-90)
GGT (IU/L)	53 (12-352)**∗∗∗**	26 (16-37)
Bilirubin (mg/dL)	0.83 (0.31-4.0)	0.76 (0.25-1.50)
INR (0.8-1.1)	1.05 (0.92-2.38)	1.1 (0.9-1.24)
Albumin (g/dL)	3.9 (2.41-4.72)**∗∗**	4.25 (3.2-5.0)
Cholesterol (mg/dL)	154.2 (140.3-231.0)	159.5 (138.0-213.0)
HDL-cholesterol (mg/dL)	41.88 (32.8-56.1)	40.58 (33.1-58.9)
Triglycerides (mg/dL)	140.5 (111.2-173.5)	118.8 (98.4-152.7)
Leucocytes (x 10^9^/L)	6.24 (3.90-12.5)	5.80 (3.90-8.90)
Platelets (x 10^9^/L)	188.5 (121.0-360.0)	190 (123.0-216.0)
Fibrosis markers		
PTX3 (ng/mL)	4.80 (1.01-12.7)**∗∗∗**	0.96 (0.2-1.96)
HA (ng/mL)	113.5 (7.9-826.9)**∗∗**	73.1 (56.4-81.8)
TGF- *β*_1_ (ng/mL)	8.0 (2.12-31.5)**∗∗∗**	2.77 (1.87-4.67)
APRI index	0.69 (0.20-12.1)**∗∗∗**	0.38 (0.16-0.47)
FIB-4 score	3.43 (0.28-30.6)**∗∗∗**	1.37 (0.38-2.54)
AST/ALT ratio	0.91 (0.45-2.70)	1.00 (0.70-2.70)
GPR ratio	0.31 (0.04-6.62)**∗∗∗**	0.13 (0.09-0.23)
LSM (kPa)	11.1±4.3	-
Histological findings		
Fibrosis stage, n (%) F 0/ 1 / 2/ 3 / 4	24 (17.4%)/ 25 (18.1 %)/ 39 (28.3 %)/ 21 (15.2 %)/ 29 (21 %)	
Liver inflammation activity stage, n (%) A 0 -1 / 2 / 3	38 (27.5%)/ 52 (37.7%)/ 48 (34.8%)	

Continuous variables are expressed as median (interquartile range, IQR) or mean ± standard deviation and categorical variables as number (percentage). Significance between groups.

^*∗*^ *P* < 0.05, ^*∗∗*^*P* < 0.01, and ^*∗∗∗*^*P* < 0.001 *versus* healthy controls. APRI, aspartate aminotransferase (AST) to platelet index; GPR, gamma-glutamyltranspeptidase to platelet ratio; HA, hyaluronic acid; LSM, liver stiffness measurement; TGF-*β*_1_, transforming growth factor‐*β*_1_.

**Table 2:** Univariate ordered logistic regression analysis with METAVIR fibrosis score as the dependent variable in patients with chronic HCV.

Variable	Coefficient (95% CI)	Standard error	Wald	*P* value
log_e_ [PTX3 (ng/mL)]	1.945 (1.772-2.123)	0.238	66.78	< 0.01
log_e_ [TGF-*β*_1_ (pg/mL)]	2.803 (2.517-3.008)	0.286	96.06	< 0.01
log_e_ [Platelets (x 10^9^/L)]	0.982 (0.967- 1.008)	0.180	29.76	< 0.01
log_e_ [GGT (IU/L)]	1.245 (1.117-1.366)	0.248	25.20	< 0.01
log_e_ [HA (ng/mL)]	1.312 (1.136-1.488)	0.321	16.70	< 0.01
log_e_ [ALT (IU/L)]	1.016 (1.004-1.034)	0.360	8.667	< 0.05
log_e_ [AST (IU/L)]	1.034 (1.014-1.116)	0.412	6.300	< 0.05
log_e_ [INR]	1.033 (1.025-1.048)	0.363	8.101	< 0.05
log_e_ [Bilirubin (mg/dL)]	1.042 (1.029-1.062)	0.382	7.442	> 0.1
log_e_ [Albumin (g/dL)]	1.062 (1.033-1.117)	0.681	2.430	> 0.1
log_e_ [Age (years)]	1.099 (0.885-1.455)	0.331	11.022	< 0.01

Data were analyzed by use of ordered logistic regression analysis. CI, confidence interval; HA, hyaluronic acid; INR, normalized international ratio; OR, odds ratio; TGF-*β*_1_, transforming growth factor‐*β*_1_.

**Table 3:** PTX3 values for the detection of significant (F≥2), advanced (F≥3) fibrosis, and cirrhosis (F=4) in patients with chronic HCV.

	Cut-off values (ng/mL)	AUC (95%CI)	Sensitivity (%)	Specificity (%)	PPV (%)	NPV (%)
F≥2	4.48	0.802 (0.727-0.877)	73.0	75.5	84.4	60.7
F≥3	5.23	0.867 (0.789-0.945 )	76.8	88.5	91.6	70.1
F=4	6.38	0.937 (0.895-0.979)	91.1	96.8	97.9	87.1

The optimal cut-off value was calculated from the ROC analysis for PTX3 and subsequently the sensitivity, specificity, positive predictive value (PPV), and negative predictive value (NPV) of the PTX3 were calculated.

**Table 4:** Comparison of PTX3 with other indicators for the diagnosis of significant fibrosis (F≥2) by areas under the receiver operating curves (ROC).

	Cut-off values	AUC (95%CI)	Sensitivity (%)	Specificity (%)	PPV (%)	NPV (%)	*P* value
PTX3 (ng/mL)	4.48	0.802 (0.727-0.877)	73.0	75.5	84.4	60.7	Reference
TGF-*β*_1_ (ng/mL)	5.77	0.943 (0.902-0.983)	91.0	98.0	98.8	85.7	< 0.01
HA (ng/mL)	69.37	0.891 (0.829-0.953)	100	73.5	87.3	100	> 0.05
APRI index	0.63	0.831 (0.756-0.906)	80.9	77.6	86.7	69.1	> 0.05
FIB‐4 score	1.86	0.770 (0.690 – 0.851)	77.5	65.3	80.2	61.5	> 0.05
GPR ratio	0.38	0.648 (0.556-0.739)	49.4	89.8	89.8	49.4	< 0.01
LSM (kPa)	8.7	0.904 (0.856-0.953)	76.4	93.9	95.8	68.7	0.02

The optimal cut-off value was calculated from the ROC analysis for PTX3, TGF-*β*_1_, HA, APRI index, FIB‐4 score, and GPR ratio and subsequently the sensitivity, specificity, positive predictive value (PPV), and negative predictive value (NPV) of the markers were calculated. APRI, platelet ratio index; FIB‐4 score; GPR, gamma-glutamyltranspeptidase to platelet ratio; HA, hyaluronic acid; TGF-*β*_1_, transforming growth factor‐*β*_1_.

## Data Availability

The data used to support the findings of this study are available from the corresponding author upon request.
